# Dr. Thomas Davison Crothers

**Published:** 1918-04

**Authors:** 


**Dr. Thomas Davison Crothers,** Albany 1865, died Jan. 12, from arterio-sclerosis, at his home in Hartford, Ct. He was born Sept. 21, 1842, at W. Charlton, N. Y., and received his pre-medical education at the Ft. Edward Seminary. After graduation, he practiced 4 years at W. Galway, N. Y. He then returned to Albany and was Asst. Prof. of Medicine in the Albany Medical College for several years. In 1875, he began institutional work as Asst. Physician in the Binghamton Inebriate Asylum. In 1878, he became Superintendent of the Walnut Hill Asylum of Hartford and, in 1880, assumed charge of the Walnut Lodge, which he continued till his death. He established the Am. Journal of Inebriety and continued in its editorial conduct till his death. He was a pronounced advocate of total abstinence and a scientific student of alcholism and other addictions. He contributed extensively to the literature of this subject, several of his articles appearing in this journal. In 1896, at a meeting of the Keuka Medical Assn., we first became personally acquainted with Dr. Crothers and enjoyed a swim together. Perhaps this circumstance gives us the feeling of having known Dr. Crothers from boyhood, his vigor and youthful enjoyment of the sport impressing us greatly at the time. We shall miss Dr. Crothers in every way, personally and professionally.

